# Effect of acupuncture on brain regions modulation of mild cognitive impairment: A meta-analysis of functional magnetic resonance imaging studies

**DOI:** 10.3389/fnagi.2022.914049

**Published:** 2022-09-23

**Authors:** Shiqi Ma, Haipeng Huang, Zhen Zhong, Haizhu Zheng, Mengyuan Li, Lin Yao, Bin Yu, Hongfeng Wang

**Affiliations:** ^1^College of Acupuncture and Massage, Changchun University of Chinese Medicine, Changchun, China; ^2^Northeast Asian Institute of Traditional Chinese Medicine, Changchun University of Chinese Medicine, Changchun, China; ^3^College of Traditional Chinese Medicine, Changchun University of Chinese Medicine, Changchun, China

**Keywords:** mild cognitive impairment, acupuncture, meta-analysis, brain regions modulation, functional magnetic resonance imaging

## Abstract

**Background:**

As a non-pharmacological therapy, acupuncture has significant efficacy in treating Mild Cognitive Impairment (MCI) compared to pharmacological therapies. In recent years, advances in neuroimaging techniques have provided new perspectives to elucidate the central mechanisms of acupuncture for MCI. Many acupuncture brain imaging studies have found significant improvements in brain function after acupuncture treatment of MCI, but the underlying mechanisms of brain regions modulation are unclear.

**Objective:**

A meta-analysis of functional magnetic resonance imaging studies of MCI patients treated with acupuncture was conducted to summarize the effects of acupuncture on the modulation of MCI brain regions from a neuroimaging perspective.

**Methods:**

Using acupuncture, neuroimaging, magnetic resonance, and Mild Cognitive Impairment as search terms, PubMed, EMBASE, Web of Science, Cochrane Library, Cochrane Database of Systematic Reviews, Cochrane Database of Abstracts of Reviews of Effects (DARE), Google Scholar, China National Knowledge Infrastructure (CNKI), China Biology Medicine disk (CBM disk), Wanfang and Chinese Scientific Journal Database (VIP) for brain imaging studies on acupuncture on MCI published up to April 2022. Voxel-based neuroimaging meta-analysis of fMRI data was performed using voxel-based d Mapping with Permutation of Subject Images (SDM-PSI), allowing for Family-Wise Error Rate (FWER) correction correction for correction multiple comparisons of results. Subgroup analysis was used to compare the differences in brain regions between the acupuncture treatment group and other control groups. Meta-regression was used to explore demographic information and altered cognitive function effects on brain imaging outcomes. Linear models were drawn using MATLAB 2017a, and visual graphs for quality evaluation were produced using R software and RStudio software.

**Results:**

A total of seven studies met the inclusion criteria, with 94 patients in the treatment group and 112 patients in the control group. All studies were analyzed using the regional homogeneity (ReHo) method. The experimental design of fMRI included six task state studies and one resting-state study. The meta-analysis showed that MCI patients had enhanced activity in the right insula, left anterior cingulate/paracingulate gyri, right thalamus, right middle frontal gyrus, right median cingulate/paracingulate gyri, and right middle temporal gyrus brain regions after acupuncture treatment. Further analysis of RCT and longitudinal studies showed that Reho values were significantly elevated in two brain regions, the left anterior cingulate/paracingulate gyrus and the right insula, after acupuncture. The MCI group showed stronger activity in the right supramarginal gyrus after acupuncture treatment compared to healthy controls. Meta-regression analysis showed that the right anterior thalamic projection ReHo index was significantly correlated with the MMSE score after acupuncture treatment in all MCI patients.

**Conclusions:**

Acupuncture therapy has a modulating effect on the brain regions of MCI patients. However, due to the inadequate experimental design of neuroimaging studies, multi-center neuroimaging studies with large samples are needed better to understand the potential neuroimaging mechanisms of acupuncture for MCI. In addition, machine learning algorithm-based predictive models for evaluating the efficacy of acupuncture for MCI may become a focus of future research.

**Systematic review registration:**

https://www.crd.york.ac.uk/prospero/display_record.php?ID=CRD42022287826, identifier: CRD 42022287826.

## Introduction

Mild cognitive impairment (MCI) is a neurodegenerative disorder between normal aging and dementia that is most prominently characterized by the presence of mild isolated cognitive decline without significant impairment in activities of daily living. MCI is considered a pre-dementia state associated with a 10-fold increased risk of progression to dementia, severely affecting the patient's quality of life (Petersen, 2011). The first clinical feature of mild cognitive impairment is memory impairment, which can involve other specific changes in motor function, executive function, language, and visuospatial structural skills, depending on the cause or the site of brain damage (Marshall et al., 2011; Montero-Odasso et al., 2017). The current global prevalence of MCI is 6.7%, with an estimated overall prevalence of 15.5% among adults aged 60 years and older in China (Petersen et al., 2018; Jia et al., 2020). MCI has become an important issue in public health and has attracted the attention of a growing number of researchers, policy makers and healthcare providers.

The main pathological mechanisms of MCI are related to amyloid pathology, neurofibrillary tangle pathology, neuronal deficits, and damage to synaptic plasticity in the hippocampal region (Kordower et al., 2001). Among these, brain amyloid-beta (Aβ) plaques are a hallmark lesion of people with a clinical diagnosis of MCI (Mufson et al., 2012). As neuroimaging methods have proliferated in recent years, more researchers have focused on alterations in brain structure and function in amnestic mild cognitive impairment (aMCI), particularly in identifying relevant neural markers. A meta-analysis reported resting-state abnormalities in the posterior cingulate, angular gyrus, parahippocampal gyrus, fusiform gyrus, superior limbic gyrus, and middle temporal gyrus in participants with MCI (Lau et al., 2016). There are no FDA-approved drugs for the treatment of MCI, and neither cholinesterase inhibitors nor memantine is recommended for the treatment of MCI (Langa and Levine, 2014). Therefore, exploring the potential of non-pharmacological interventions to prevent MCI has received increasing attention.

As a suitable alternative medical treatment, acupuncture has been used empirically for thousands of years while gaining worldwide attention and recognition (Kim et al., 2019). Numerous previous clinical and animal studies have shown that acupuncture may be an effective adjunctive treatment for neurological disorders, such as cognitive impairment, Alzheimer's disease, and dementia, and can effectively improve cognitive and memory function (Du et al., 2018; Ding et al., 2019; Ji et al., 2021; Su et al., 2021; Zhi et al., 2021). The therapeutic mechanism may be related to downregulation of Aβ accumulation and tau protein phosphorylation, reduction of neuroinflammation, reduction of neuronal apoptosis, improvement of mitochondrial activity, enhancement of synaptic plasticity, and restoration of the blood-brain barrier (Yin et al., 2021). However, there is a lack of research to explore the therapeutic mechanisms of acupuncture for MCI from the perspective of brain region modulation. Therefore, it is necessary to explore the mechanism of action from the perspective of brain structure and function.

Numerous studies have proven that neuroimaging techniques can accurately record changes in brain regions for neurological diseases and treatment effects (Jiang et al., 2015; Dan, 2019; Risacher and Saykin, 2021). Neuroimaging methods have become a critical tool for performing research to develop a better understanding of brain circuit alterations associated with etiology, pathophysiology, and treatment response (Kalin, 2021). The spatial variability properties of the brain were evaluated by analyzing changes in regional homogeneity (ReHo) and amplitude of low-frequency fluctuations (ALFFs). An increasing number of studies have applied fMRI techniques to evaluate the clinical effects of acupuncture for MCI (Wang et al., 2012; Liu et al., 2014; Shan et al., 2018). However, the small sample size between the different clinical designs led to variability in the experimental results.

Therefore, to elucidate the modulatory effects of brain regions in acupuncture for MCI, this paper uses a coordinate-based meta-analysis (CBMA) to integrate the imaging findings from clinical studies quantitatively. The CBMA is a widely used method to solve the discrepancies of regional alterations among various neuroimaging studies (Jiang et al., 2017). The Seed-based d Mapping with Permutation of Subject Images (SDM-PSI) is an advanced statistical technique for CBMA on different neuroimaging techniques such as structural MRI, fMRI, DTI, or PET (Albajes-Eizagirre et al., 2019a). The SDM-PSI approach allows reported peak coordinates combined with statistical parametric maps, thus ensuring more exhaustive and accurate meta-analyses (Albajes-Eizagirre et al., 2019c). By analyzing the effect of acupuncture on the modulation of MCI brain regions, the therapeutic effect of acupuncture was elucidated from a neuroimaging perspective, providing new ideas for treating neurological diseases with acupuncture.

## Materials and methods

All procedures for this meta-analysis were performed following the Preferred Reporting Items for Systematic Reviews and Meta-Analyses guidelines (PRISMA guidelines) (PRISMA). This study was registered on the International Prospective Register of Systematic Reviews (PROSPERO: CRD 42022287826) (Liberati et al., 2009; Moher et al., 2009; Page et al., 2021).

### Literature search

We searched the following electronic databases from the establishment of the databases to April 2022: PubMed, EMBASE, Web of Science, Cochrane Library, Cochrane Database of Systematic Reviews, Cochrane Database of Abstracts of Reviews of Effects (DARE), Google Scholar, China National Knowledge Infrastructure (CNKI), China Biology Medicine disk (CBM disk), Wan Fang, and Chinese Scientific Journal Database (VIP). The search process was carried out independently by two researchers. The search terms included: (mild cognitive impairment OR cognitive impairment OR cognitive decline OR cognitive deficit OR cognitive dysfunction OR cognitive disorders OR cognitive dissonance OR amnestic OR MCI) AND (acupuncture OR meridian OR acupuncture therapy OR acupuncture treatment OR acupoint OR electroacupuncture OR electro-acupuncture OR ear acupuncture OR auriculotherapy OR scalp acupuncture) AND (RCT OR randomized controlled trial OR controlled clinical trial OR randomized OR clinical trial OR randomly OR trial OR “random*” OR “alloc*”OR “assign*) AND (fMRI OR functional MRI OR functional magnetic resonance imaging OR neuroimaging OR voxel-based morphometry OR VBM OR resting state). In addition, professional journals, reference lists of relevant articles, and conference abstracts related to MCI and acupuncture were hand-searched in the library to ensure a comprehensive literature search. Among them, four Chinese journals and two English journals related to acupuncture were manually searched in the library:Acupuncture Research (from 1976), Chinese Acupuncture and Moxibustion (from 1981), Journal of Clinical Acupuncture and Moxibustion (from 1985), Shanghai Journal of Acupuncture and Moxibustion (from 1982), Acupuncture in Medicine (from 1982), and Medical Acupuncture (from 2007) through March 2017. The language during the search process is restricted to articles published in English or Chinese. Boolean logic operations are used to develop search formulas for different search libraries.

### Inclusion and exclusion criteria

All studies were screened for title, abstract and full text and were conducted independently by two researchers (ML and ZZ). In case of disagreement, the two researchers reached an agreed result through discussion. The following inclusion criteria were based on PICO standards:

Participants: Clinical trials with clear diagnostic criteria for MCI, with no restrictions on participant age or gender.

(1) Interventions: The treatment group used various acupuncture therapies (e.g., pure acupuncture, body acupuncture, electroacupuncture, ear acupuncture) or acupuncture combined with other medications. We did not set limitations for intensity, frequency, or course of treatment.
(2) Comparison Groups: The comparison group can be treated with any non-acupuncture method but should be consistent with the baseline information of the intervention group (e.g., age, gender, etc.).
(3) Outcomes: Functional magnetic resonance imaging (fMRI) and subjective scale outputs. It mainly involves whole-brain functional imaging (ReHo or ALFFs) at rest or in the task state. Peak coordinates (*x, y, z*) and effect sizes (*t*-value, or *z*-value or *P*-value) reported in Talairach or Montreal Neurological Institute (MNI) standard stereotactic space. All samples were included if one study involved two or more comparable datasets. Secondary outcomes were used to assess clinical efficacy, measured using the Clinical Dementia Rating (CDR) and the Brief Mental State Examination (MMSE).

We excluded the following types of articles: articles using ROI or seed voxel-based analyses, missing significant information on results [e.g., coordinates significant clusters (*P* < 0.05)], methodological studies, conference summaries, and preliminary trials with complete overlap.

### Study selection

Two independent evaluators (ZZ and HZ) screened the literature based on inclusion criteria. Titles and abstracts of all studies retrieved through the search strategy were first screened using EndNote and duplicates were removed. The second screening was performed mainly by further review of the full text of the literature. In case of disagreement during the screening process, the document was submitted to a third evaluator for consultation and eventual agreement.

### Data extraction

We extracted the full text of the literature based on a pretest post data extraction form. Data extraction was performed independently by two assessors (LY and YL) based on inclusion and exclusion criteria, followed by cross-checking. Data were validated by a third assessor (HH). If data were missing, authors were contacted by email for further information. Data were extracted from the included studies with the following standardization: (1) publication data (author, year); (2) basic information about the trial design study (Study trial type, Comparison, Sample size, Scanning instrument, clinical outcome measures, neuroimaging techniques, task-based/resting-state study design, episodic/interval conditions, image acquisition timing, analysis methods); (3) acupuncture manipulation (primary acupoints, acupuncture modality, frequency, duration, duration of treatment); (4) participants (gender, age, education, Symptom severity); (5) neuroimaging results.

### Quality assessment

Quality assessment was based on the Cochrane Risk of Bias tool and was conducted independently by two researchers. All reports were assessed according to the following seven criteria: random sequence generation, allocation concealment, blinding of participants and personnel, blinding of outcome assessment, incomplete outcome data, selective reporting, and other sources of bias. For each criterion, studies were judged to be at low, high, or unclear risk of bias. Visual graph production for quality evaluation using R software version 4.1.3 and R Studio version 2022.02.0.

### SDM-PSI meta-analysis

Voxel-based meta-analyses of regional brain differences were performed using Seed-based d Mapping with Permutation of Subject Images (SDM-PSI) (version 6.21, https://www.sdmproject.com/). This software package uses reported peak coordinates extracted from databases with statistical parametric maps. It reconstructed the original maps of regional differences in the brain, thus revealing the neural substrates of many brain functions and neuropsychiatric disorders (Radua et al., 2012; Albajes-Eizagirre and Radua, 2018). The procedures included collecting the data, creating SDM table, pre-processing, mean analysis, heterogeneity, publication bias, and grading.

In the data collection step, a text file is created for each study, containing the peak coordinates and *t*-values, and the name of the text file must be “XXX.spm_mni.txt.” If the study had no peaks, its text file was recorded as having no content with the extension “.no_peaks.txt”. In the Create SDM Table step, enter general information about the studies in the SDM Table Editor, including their identification (column “study”), their sample sizes, the *t*-value that they used as statistical thresholds (column “t_thr”), and other potential variables to conduct subgroup analyses or meta-regressions. Of particular note is the presence of specified thresholds in each study, applying the same statistical thresholds to estimate the maps more accurately. In the preprocessing step, SDM-PSI estimates the lower limit of the size of the possible effect size images (i.e., the lowest potential effect size for each voxel) and their upper limit (i.e., the maximum potential effect size for each voxel) of the images from the peak coordinates and effect sizes collected for each study, respectively, to compare the peak coordinates and effect sizes between the treatment and control groups. SDM-PSI performed a meta-analysis of NSUE (MetaNSUE) based on maximum likelihood estimation and multiple imputation algorithms (Radua et al., 2012; Albajes-Eizagirre et al., 2019b). MetaNSUE was used to estimate the most likely effect sizes and their standard errors, thus creating several imputations. In the mean analysis, the weighted mean difference of the regional gray matter of the sample size of this study is expressed. This includes calculating the random-effects mean of the ReHo values, with the mean weighted by the sample size and variance of each study. Secondly, a meta-analysis was performed for each dataset using a standard random-effects model, and then the coefficients of these datasets and their covariances and heterogeneity statistics I and Q were combined using Rubin's rule. Finally, corrections were made by clustering-based thresholds, using uncorrected *p* < 0.001 as the threshold for cluster formation at the cluster level, along with Family-Wise Error Rate (FWER) correction (*p* < 0.05 and voxel extent ≥ 10) and the use of threshold-free clustering enhancement (TFCE) in statistical thresholds at the cluster level. To set the null distribution, 1000 replacement trials were performed. The details of these procedures are extensively described in the SDM-PSI reference manual (https://www.sdmproject.com/manual/).

### Heterogeneity and publication bias

The MNI peak coordinates were extracted and analyzed for heterogeneity to obtain the standard heterogeneity statistic *I*^2^. *I*^2^ < 50% indicates low heterogeneity. Funnel plots were not performed because the amount of included studies (*n* = 7) was <10, but the Egger test was used to assess the publication bias.

### Meta-regression analysis

The potential effects of clinical variables such as gender, age, years of education, duration of illness, and severity of clinical symptoms (*p* < 0.00005, uncorrected, and voxels > 10 indicate statistical differences) were explored by simple linear regression analysis. Linear models were drawn using MATLAB 2017a.

## Results

### Characteristics of included studies

**Figure 1:**
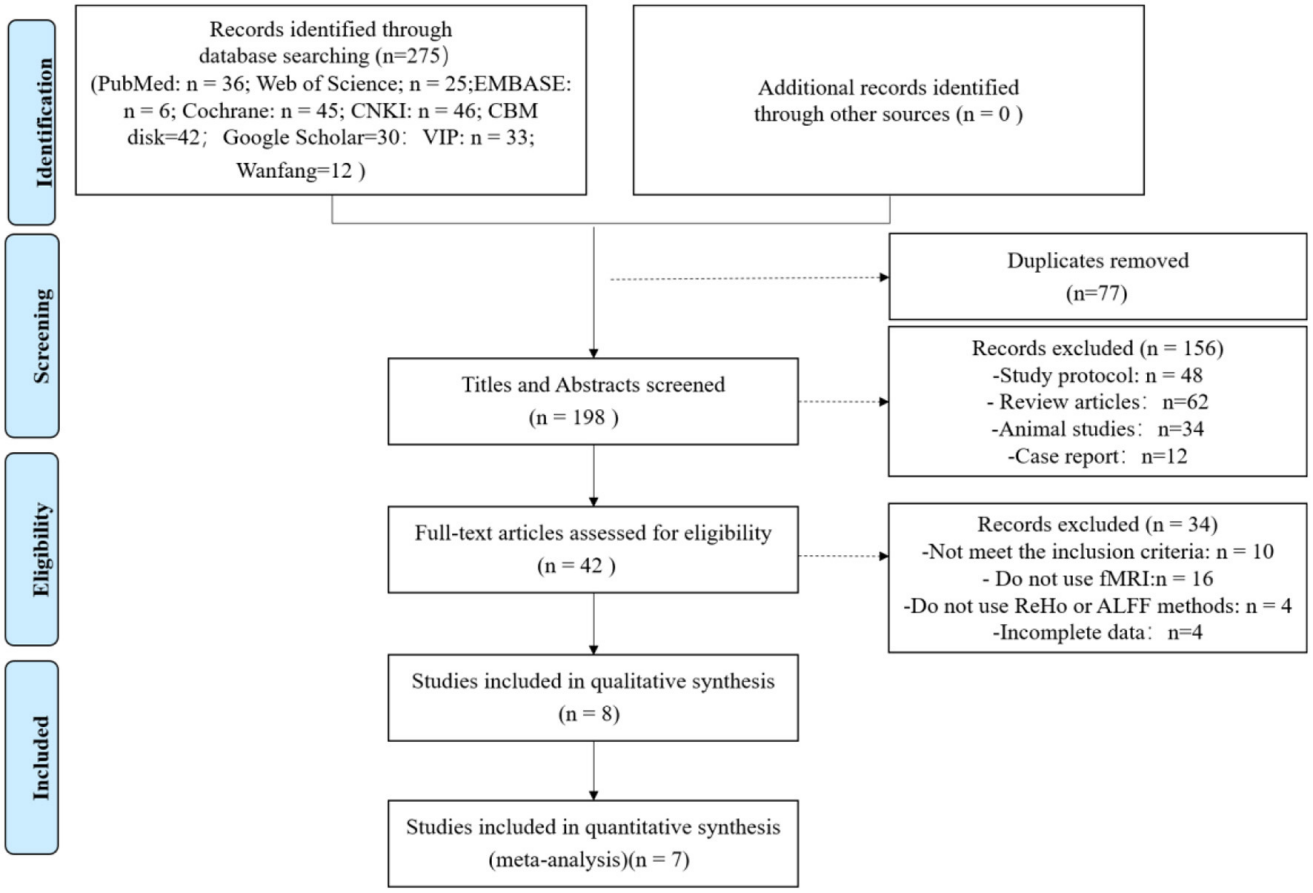
Flowchart of literature selection.

Our search identified seven studies that met the inclusion criteria (Hou et al., 2010; Jiang et al., 2012; Wang et al., 2012, 2020; Liu et al., 2014; Jia et al., 2015; Shan et al., 2018). Based on the search strategy, the database search identified 275 articles and 77 articles were deleted due to duplication. After screening by title and abstract, 156 articles were further excluded. Of the 42 eligible relevant studies, 34 studies were excluded after the full-text screening. Of these 34 studies, 10 articles did not meet the inclusion criteria, 16 did not use fMRI, four did not use ReHo or ALFF methods, and four had incomplete data. A total of seven studies were included in the final analyses. Figure 1 represents the PRISMA flow diagram of the article search.

All trials that met the inclusion criteria were published between 2010 and 2020. All clinical studies recruited one hundred and eighty patients (112 MCI patients and 68 healthy controls). There were no significant differences in demographic baseline characteristics (including age, gender, and years of education) between the two groups. Baseline characteristics of clinical symptoms were assessed using subjective scales (including MMSE, CDR, and auditory verbal learning test), and all met the inclusion criteria. Most studies used a new non-repeated event-related (NRER) fMRI design model to explore the ongoing effects of acupuncture on MCI. Only 1 study used a conventional acupuncture modality with a 4-week duration of treatment (Wang et al., 2020). The Main acupoints included Tai Chong (LR3), Tai Xi (KI3), Bai Hui (DU20) and He Gu (LI4). The acupuncture method was mainly carried out in the balanced “tonifying and reducing” technique, while the positioning and operation of acupuncture points were based on international standards (Hui et al., 2000). All studies reported fMRI coordinate data using the ReHo variable to analyze the brain region activation effect before and after acupuncture treatment and the difference in its comparison with sham acupuncture and healthy control group. The details and features of the studies are shown in Tables 1, 2. Anatomical localization of the acupoints mentioned in the included studies are shown in Supplementary material 2.

**Table 1:** Demographic and clinical characteristics of included studies.

**References**	**Study design**	**Groups (*n*, male/female)**	**Treatments (*n*)**	**Age (years)**	**Education (years)**	**Symptom severity (baseline)**	**Main acupoints (placement, number)**	**Experimental design (parameters, session duration, total period)**
Wang et al. (2020)	Longitudinal study	MCI (36, 16/20)	MA (36)	MA: 64.96 ± 3.22	/	MMSE: 24.61 ± 1.73; MoCA: 22.79 ± 1.79	Head: DU20 (1), BG13 (2), GB20 (2);Foot: LR3 (2), SP3 (2), KI3 (2);Abdomen: RN4;Hand: HT7 (2); Leg: ST40 (2), BL58 (2)	MA: uniform reinforcing-reducing method; 40 min/time, 6 times/w, for 4 weeks.
Shan et al. (2018)	Cross sectional	MCI (14, 6/8); HC(14, 6/8)	RA: MCI(8), HC(14); SA: MCI (6)	RA: 66.38 ± 10.97; SA: 67.83 ± 6.01; HC: 66.07 ± 5.78	RA: 10.63 ± 3.54; SA: 11.00 ± 3.16; HC: 11.00 ± 4.52	•MMSE: RA: 25.38 ± 1.30, SA: 25.67 ± 2.34; HC: 28.00 ± 1.41 • CDR: RA: 0.5, SA: 0.5, HC: 0 • AVLT (immediate): RA: 14.13 ± 3.52, SA: 22.50 ± 3.02, HC: 26.86 ± 5.25 • AVLT (delayed): RA: 4.38 ± 1.60, SA: 7.83 ± 3.92, HC: 11.07 ± 2.76 • AVLT (recognition): RA: 7.38 ± 3.11, SA: 9.17 ± 3.19, HC: 12.71 ± 2.09	Foot: LR3 (2); Hand: LI4 (2)	MA: needles are 0.3 mm in diameter, 25 mm long and 2 cm deep;rotated continuously (±180°, 60 times/min); 3 min; SA: 10 mm next to LR3 and Hegu; SA and HC needle specifications and treatment time are the same as MA.
Jia et al. (2015)	Cross sectional	MCI (8, 2/6); HC (15, 8/7)	RA: MCI (8); SA: MCI (8); HC (15)	MCI: 74.1 ± 7.8; HC: 70.2 ± 7.1	MCI: 12.5 ± 3; HC: 11.4 ± 4.2	•MMSE: MCI: 27.0 ± 2.3; HC: 29.2 ± 1.3 • ADAS-cog: MCI: 6.7 ± 2.9; HC: 2.5 ± 1.7	Foot: KI3 (2)	RA: needles are 0.25 mm in diameter, 40 mm long and 2 cm deep 1 min, continuous rotation, right then left, at a frequency of 2 Hz for 60 s; SA: 25 mm directly above KI3 as a sham control, and the rest was the same as RA.
Liu et al. (2014)	Cross sectional	MCI (12, 1/11); HC(12, 4/8)	MA: MCI (12), HC (12)	MCI: 59.3 ± 3.3; HC: 60.6 ± 5.8	MCI: 10.5 ± 1.8; HC: 10.6 ± 2.06	•MMSE: MCI: 26.4 ± 0.9, HC: 29.8 ± 0.4 • CDR: MCI: 0.5, HC: 0	Foot: KI3 (2)	MA: needle is 0.2 mm in diameter, 40 mm long and 1-2 cm deep; rotated continuously (±180°, 60 times/ min), 3 min; HC and MA operate in the same way.
Wang et al. (2012)	Cross sectional	MCI (8, 3/5); HC (14, 6/8)	MA: MCI (8); HC (14)	MCI: 66.37 ± 10.9; HC: 66.07 ± 5.78	/	•MMSE; MCI: 25.37 ± 1.30, HC: 28.00 ± 1.41 • AVLT (immediate): MCI: 14.13 ± 3.52, HC: 26.86 ± 5.24 • AVLT (delayed): MCI: 4.37 ± 1.59, HC: 11.07 ± 2.76 • AVLT (recognition): MCI: 7.38 ± 3.11;HC: 12.71 ± 2.09 • CDR: MCI: 0.5, HC: 0	Foot: LR3 (2); Hand: LI4 (2)	MA: 3 min; HC: None
Jiang et al. (2012)	RCT	MCI (24, 12/12)	RA: MCI (12); SA: MCI (12)	MA: 63.83 ± 4.90; SA: 67.08 ± 5.26	/	CDR: 0.5, MMSE ≥ 24	Foot: LR3 (2)	MA: Needle diameter of 0.35 mm, 25 mm long, 1.5 cm deep; uniform lifting and inserting twisting row needle method, lifting and inserting amplitude in the upper and lower 2–3 mm, 30 min; SA: The midpoint of the line between the right KI3 point and the Achilles tendon.
Hou et al. (2010)	Cross sectional	MCI (10); HC (13)	EA: MCI (10); HC: 13	50–80	/	MMSE ≥ 24, MoCA < 26	Head: DU20 (1), EX-HN1 (4), BG13 (2); Leg: ST36 (2), ST40 (2), SP6 (2)	EA: Needle diameter of 0.30 mm, 40 mm long, 1.5 cm deep; rotated continuously (±180°, 120 times/min), Sparse and dense waves, 2 min

**Table 2:** Scanning methods and major brain region alterations in the included studies.

**References**	**Scanning instrument/ experimental design**	**Seed regions**	**Analysis of fMRI**	**Statistical threshold**	**Number of coordinates**	**Main conclusions**
Wang et al. (2020)	Achieva 3.0 T; RS	Whole brain	Reho	*P* = 0.001, uncorrected	MNI, 7	Acu vs. HC: right parahippocampal gyrus, left thalamus, right insula, and left anterior cingulate gyrus↑; left posterior cerebellar lobe, left inferior temporal gyrus, right inferior temporal gyrus, left inferior frontal gyrus, left middle temporal gyrus, left inferior occipital gyrus, and left superior parietal lobule↓
Shan et al. (2018)	Siemens3.0T; NRER	Whole brain	Reho	*P* < 0.05, AlphaSim corrected	MNI, 11	• Acu vs. Sham: left supramarginal gyrus, left superior temporal gyrus, left rolandic operculum, left cerebellum, right middle frontal gyrus, and right inferior frontal gyrus (pars opercularis) ↑; left inferior parietal gyrus↓ • Acu vs. HC: right superior temporal gyrus, right superior temporal gyrus, right superior parietal gyrus, right supramarginal gyrus, right postcentral gyrus, right precentral gyrus, right cerebellum, left inferior parietal gyrus, left middle occipital gyrus, and left inferior occipital gyrus↑
Jia et al. (2015)	Tesla Signa (GE) MR 1.5 T;NRER	Whole brain	Reho	*P* < 0.01, AlphaSim corrected	MNI, 3	Acu vs. Sham: right superior temporal gyrus↑; middle prefrontal gyrus ↓
Liu et al. (2014)	Tesla Signa (GE) MR 3.0T; NRER	Whole brain	Reho	*P* < 0.01, AlphaSim corrected	MNI, 13	Acu vs. HC: MTG, superior parietal lobule (SPL), middle frontal gyrus (MFG), superior marginal gyrus (SMG), and PCG↑
Wang et al. (2012)	Siemens3.0T; NRER	Whole brain	Reho	*P* < 0.001, uncorrected	MNI, 44	• AS vs. RS(First): bilateral cerebellum posterior lobe, temporal lobe, frontal lobe, parietal lobe and occipital lobe↑; bilateral CPL, temporal lobe, frontal lobe, parietal lobe right lingual gyrus and limbic regions↓ • AS vs. RS(Second):bilateral CPL, temporal lobe, frontal lobe, right lentiform nucleus, left extra nuclear and right thalamus↑; bilateral CPL, temporal lobe, frontal lobe, parietal lobe and occipital lobe↓
Jiang et al. (2012)	Achieva 3.0T; NRER	Whole brain	Reho	*P* < 0.001, uncorrected	MNI,5	Acu vs. Sham: right cingulate gyrus, bilateral medial frontal gyrus and left postcentral gyrus↑
Hou et al. (2010)	Achieva 1.5T; NRER	Whole brain	Reho	*P* < 0.001, uncorrected	MNI, 6	Acu vs. HC: superior temporal gyrus in the posterior temporal lobe, orbitofrontal and frontopolar regions of the frontal lobe, and temporal cortex↑

RS, resting state; AS, acupuncture stste; NRER, non-repeated event-related; ReHo: regional homogeneity; MNI: Montreal Neurological Institute.

### Quality assessment

**Figure 2:**
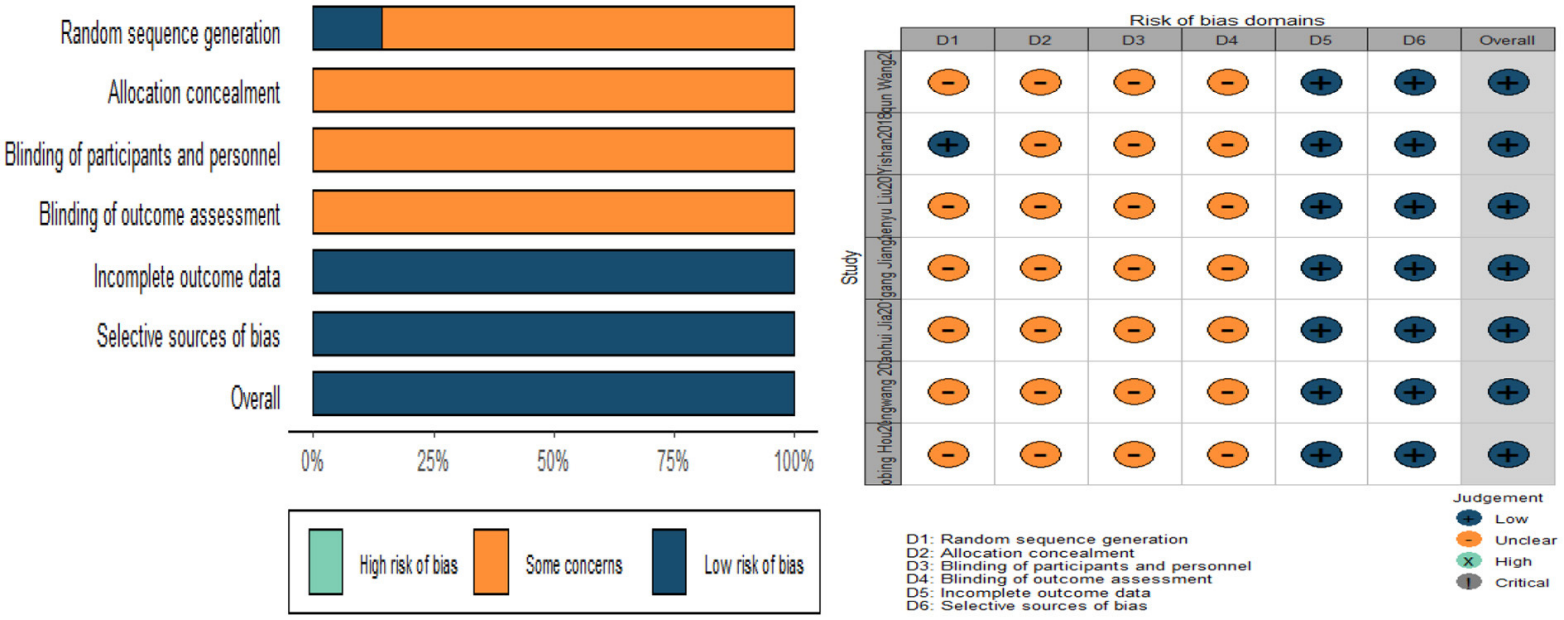
Quality assessment of included studies.

The quality assessment was performed using the Cochrane Risk of Bias tool, and the evaluation criteria were divided into seven entries. Of the seven studies, only one reported on the method of random sequence generation (Shan et al., 2018). No studies mentioned allocation concealment and blinding, and the risk of bias was unclear. Evaluation of incomplete outcome data depended on whether the clear descriptions of baseline data were shown. Based on this evaluation criterion, all studies reported outcome data in full and were judged to be low risk. Although none of the studies had a study protocol, all expected outcome indicators were reported, including those that were predetermined and therefore judged to be low risk. In addition, we did not find any other sources of bias. In general, the quality of these studies was not high, mainly in terms of study design. Figure 2 illustrates the quality assessment of the included studies.

### Results of meta-analysis

#### Acupuncture for the modulation of brain regions in MCI patients

**Figure 3:**
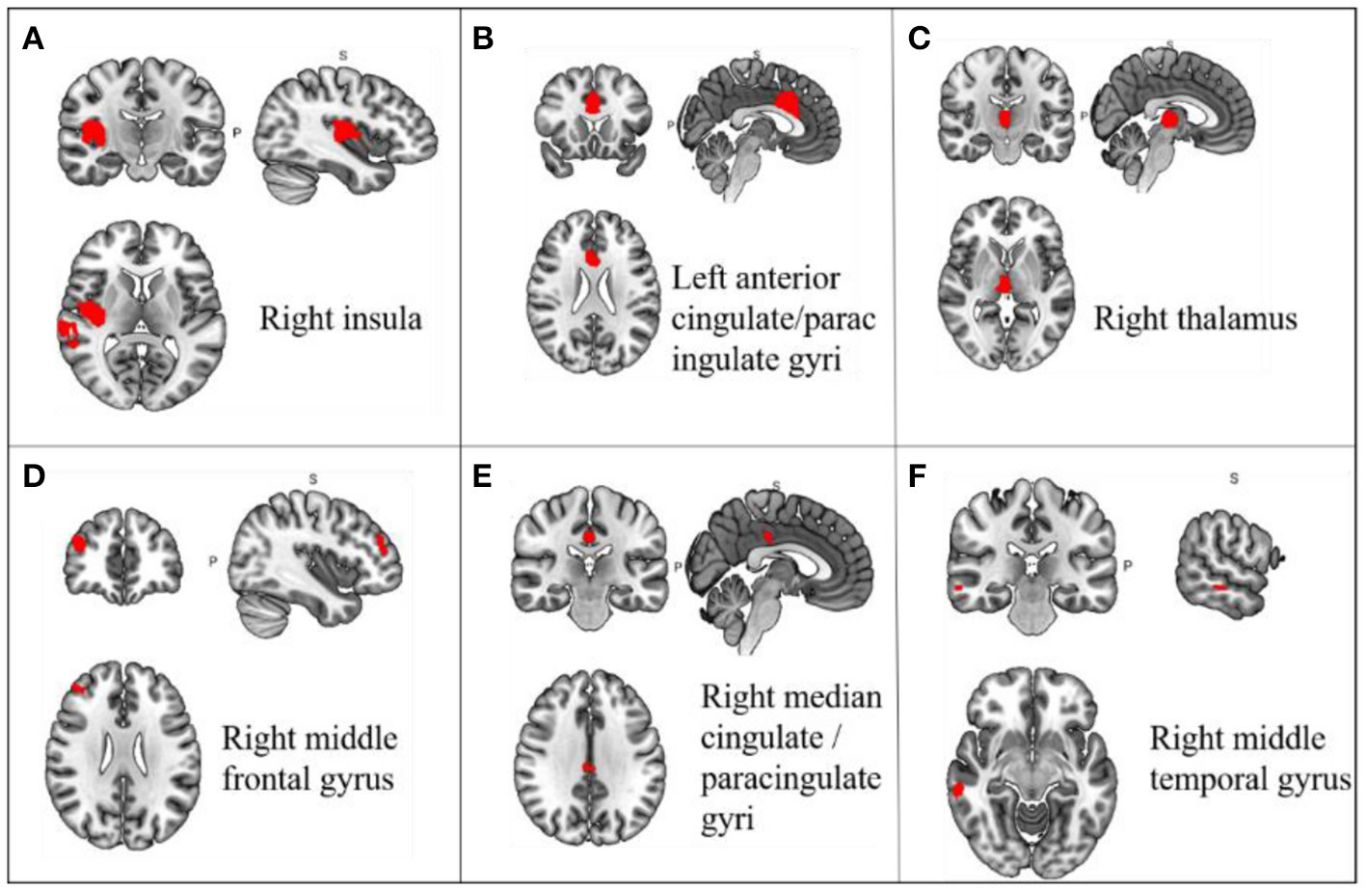
Changes in gray matter regions PRE and POST acupuncture treatment in MCI patients. **(A)** Right insula; **(B)** Left anterior cingulate/paracingulate gyri; **(C)** Right thalamus; **(D)** Right middle frontal gyrus; **(E)** Right median cingulate/paracingulate gyri; **(F)** Right middle temporal gyrus. Important clusters are presented with MRIcron templates.

In a pooled meta-analysis, brain region coordinates based on Family-Wise Error Rate (FWER) correction (*p* < 0.05) thresholds were analyzed for PRE and POST acupuncture treatment for MCI patients in the group. The results showed that acupuncture treatment showed significant increases in Reho values in six brain regions, mainly including the right insula (*p* < 0.05, *z* = 3.362), left anterior cingulate/paracingulate gyri (*p* < 0.05, *z* = 3.482), right thalamus (*p* < 0.05, *z* = 3.967), right middle frontal gyrus (*p* < 0.05, *z* = 2.544), right median cingulate/paracingulate gyri (*p* < 0.05, *z* = 2.185) and right middle temporal gyrus (*p* < 0.05, *z* = 2.332), indicating hyperactivation of these brain regions after acupuncture treatment. The differences in regional activity in the gray matter of the brain PRE and POST acupuncture treatment in MCI patients based on coordinate analysis are shown in Table 3 and Figure 3. Further analysis of the RCTs and longitudinal studies (Jiang et al., 2012; Wang et al., 2020) revealed significantly higher Reho values in two brain regions after acupuncture, including the left anterior cingulate/paracingulate gyrus (*p* < 0.05, *z* = 3.482) and the right insula (*p* < 0.05, *z* = 3.362) (see Table 4).

**Table 3:** Regional differences in brain gray matter volume activity PRE and POST acupuncture treatment in the coordinate-based meta-analysis.

**Brain regions**	**MNI coordinates**			**SDM Value**	***p*-value**	**Voxels**	**Cluster breakdown (number of voxels)**	**I^2^**
	***x***	***y***	***z***					
Right insula, BA 48	40	−18	8	3.362	0.000386775	1,342	• Right insula, BA 48 (315); Right superior temporal gyrus, BA 21, 22, 42, 48 (360); Right rolandic operculum, BA 48 (148) • Corpus callosum (119) • Right heschl gyrus, BA 48 (106) • Right middle temporal gyrus, BA 21, 22 (130) • Right lenticular nucleus, putamen, BA 48 (50) • Right fronto-insular tract 5 (23) • Right supramarginal gyrus, BA 48 (13)	1.01%
Left anterior cingulate/paracingulate gyri	0	14	26	3.482	0.000248611	748	• Left median cingulate/paracingulate gyri, BA 24 (164) • Right median cingulate/paracingulate gyri, BA 24, 32 (179) • Right median network, cingulum (121) • Right anterior cingulate/paracingulate gyri, BA 24 (141) • Left anterior cingulate/paracingulate gyri( 64) • Corpus callosum (53) • Left superior frontal gyrus, medial, BA 32 (18)	0.95%
Right thalamus	4	−18	4	3.967	0.000036418	349	• Right thalamus (130) • Left thalamus (60) • Right anterior thalamic projections (27)	0.64%
Right middle frontal gyrus, BA 46	42	48	18	2.544	0.005474687	138	• Right middle frontal gyrus, BA 46 (78) • Right middle frontal gyrus, BA 45 (51)	17.23%
Right median cingulate/paracingulate gyri, BA 23	4	−24	34	2.185	0.014458418	83	• Right median cingulate/paracingulate gyri, BA 23 (42) • Left median cingulate/paracingulate gyri, BA 23 (32)	1.34%
Right middle temporal gyrus, BA 21	64	−26	−12	2.332	0.009853780	45	• Right middle temporal gyrus, BA 21 (31) • Right middle temporal gyrus, BA 20 (12)	5.90%

Peak height threshold: z > 1. Voxel probability threshold: P < 0.005 uncorrected and remained after correcting threshold (TFCE) of P < 0.05. Cluster extent threshold: number ≥ 10 voxels. BA, Brodmann area; I^2^, heterogeneity I^2^; MNI, Montreal Neurological Institute; R, right; ReHo, regional homogeneity; SDM, signed differential mapping.

**Table 4:** Regional differences in brain gray matter volume activity before and after acupuncture treatment in cross-sectional and longitudinal studies.

**Brain regions**	**MNI coordinates**			**SDM Value**	***p*-value**	**Voxels**	**Cluster breakdown (number of voxels)**	**I^2^**
	***x***	***y***	***z***					
Left anterior cingulate/paracingulate gyri	0	14	26	3.482	0.029999971	51	• Right anterior cingulate/paracingulate gyri, BA 24 (24) • Left anterior cingulate/paracingulate gyri, BA 24 (14) • Right median cingulate/paracingulate gyri, BA 24 (7) • Right median network, cingulum (6) • Right heschl gyrus, BA 48 (106) • Right middle temporal gyrus, BA 21, 22 (130) • Right lenticular nucleus, putamen, BA 48 (50) • Right fronto-insular tract 5 (23) • Right supramarginal gyrus, BA 48 (13)	0.72%
Right insula, BA 48	40	−18	8	3.362	0.029999971	41	• Right insula, BA 48 (29) • Right heschl gyrus, BA 48 (11) • Corpus callosum (1)	3.75%

Peak height threshold: z > 1. Voxel probability threshold: P < 0.005 uncorrected and remained after correcting threshold (TFCE) of P < 0.05. Cluster extent threshold: number ≥ 10 voxels. BA, Brodmann area; I^2^, heterogeneity I^2^; MNI, Montreal Neurological Institute; R, right; ReHo, regional homogeneity; SDM, signed differential mapping.

EA, electroacupuncture; MA, manual acupuncture; RA, real acupuncture; MMSE, Mini-mental State Examination; MoCA, Montreal Cognitive Assessment; AVLT, auditory verbal learning test; CDR, Clinical Dementia Rating; SA, sham acupuncture; HC, healthy controls; RCT, randomized controlled trial; Min, minutes; DU20, Baihui; KI3, Taixi; GV20, Baihui; RN4, Guanyuan; LR3, Taichong; LI4, Hegu; SP6, Sanyinjiao; GB20, Fengchi; EX-HN1, Sishecong; SP3,Taibai; ST40, Fenglong; BL58, Feiyang; HT7, Shenmen; BG13, Benshen; ST36, Zusanli.

#### Differences in brain region modulation by acupuncture in MCI patients and healthy subjects

**Figure 4:**
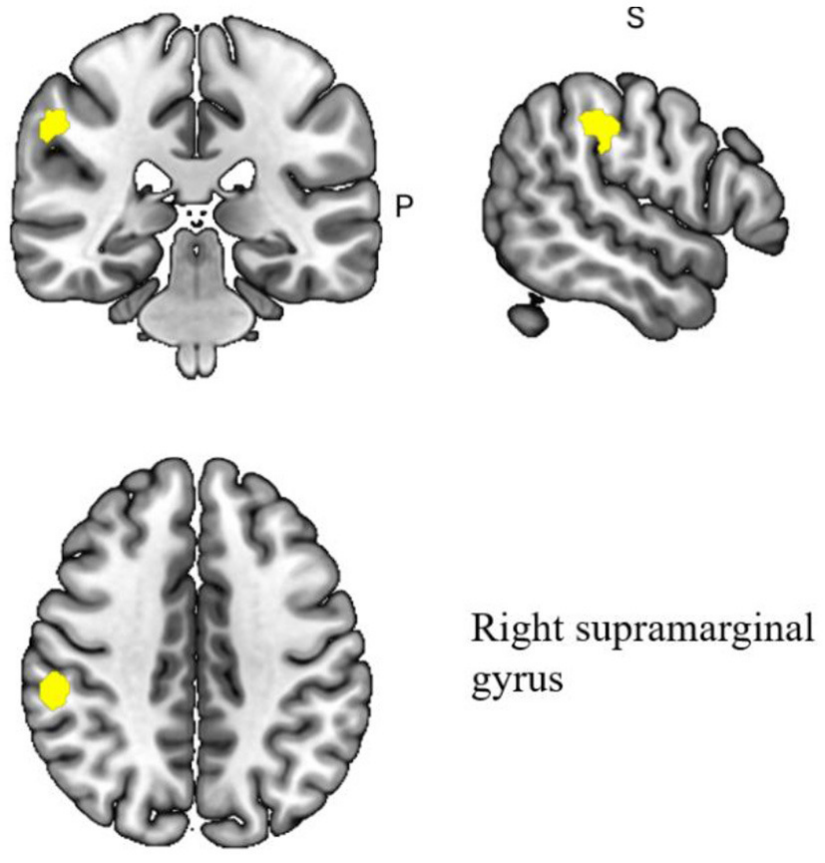
Regional differences in gray matter between MCI patients and healthy controls after acupuncture treatment.

A pooled meta-analysis analyzed differences in brain areas between the MCI and healthy control groups after acupuncture treatment and included four cross-sectional studies Hou et al., 2010; Wang et al., 2012; Liu et al., 2014; Jia et al., 2015; Shan et al., 2018. The results showed a significant difference in the right supramarginal gyrus after acupuncture treatment in the MCI group compared to the healthy control group. At the same time, the right supramarginal gyrus extended to the right postcentral gyrus and right superior longitudinal fasciculus III. The differences in the regulation of brain regions between MCI patients and healthy individuals by acupuncture are shown in Table 5 and Figure 4.

**Table 5:** Regional differences in gray matter volume after acupuncture treatment in MCI patients vs. healthy controls in a coordinate-based meta-analysis.

**Brain regions**	**MNI coordinates**			**SDM Value**	**P-value**	**Voxels**	**Cluster breakdown (number of voxels)**	**I^2^**
	***x***	***y***	***z***					
Right supramarginal gyrus, BA 2	56	−26	38	3.525	0.000999987	180	Right supramarginal gyrus, BA 2 (67); Right supramarginal gyrus, BA 40 (50); Right superior longitudinal fasciculus III (32); Right supramarginal gyrus, BA 48 (18); Right supramarginal gyrus, BA 3 (8); Right postcentral gyrus, BA 3 (3); Right postcentral gyrus, BA 2 (2)	1.92%

Peak height threshold: *z* > 1. Voxel probability threshold: *P* < 0.005 uncorrected and remained after correcting threshold (TFCE) of *P* < 0:05. Cluster extent threshold: number ≥ 10 voxels. BA, Brodmann area; *I*^2^, heterogeneity *I*^2^; MNI, Montreal Neurological Institute; R, right; ReHo, regional homogeneity; SDM, signed differential mapping.

### Heterogeneity analysis and publication bias

Heterogeneity analysis showed variability among the different studies included. In addition, we used Egger's test to assess potential publication bias in the meta-analysis. There was low heterogeneity in the peak coordinate effect size differences in the right insula, left anterior cingulate/paracingulate gyrus, right thalamus, right middle frontal gyrus, right cingulate/paracingulate gyrus, and right middle temporal gyrus (*I*^2^ = 0.64–17.23). The heterogeneity results are shown in Table 2. The Egger test differences were not statistically significant (*p* = 0.432), and the study had no significant publication bias.

### Meta regression analysis

**Figure 5:**
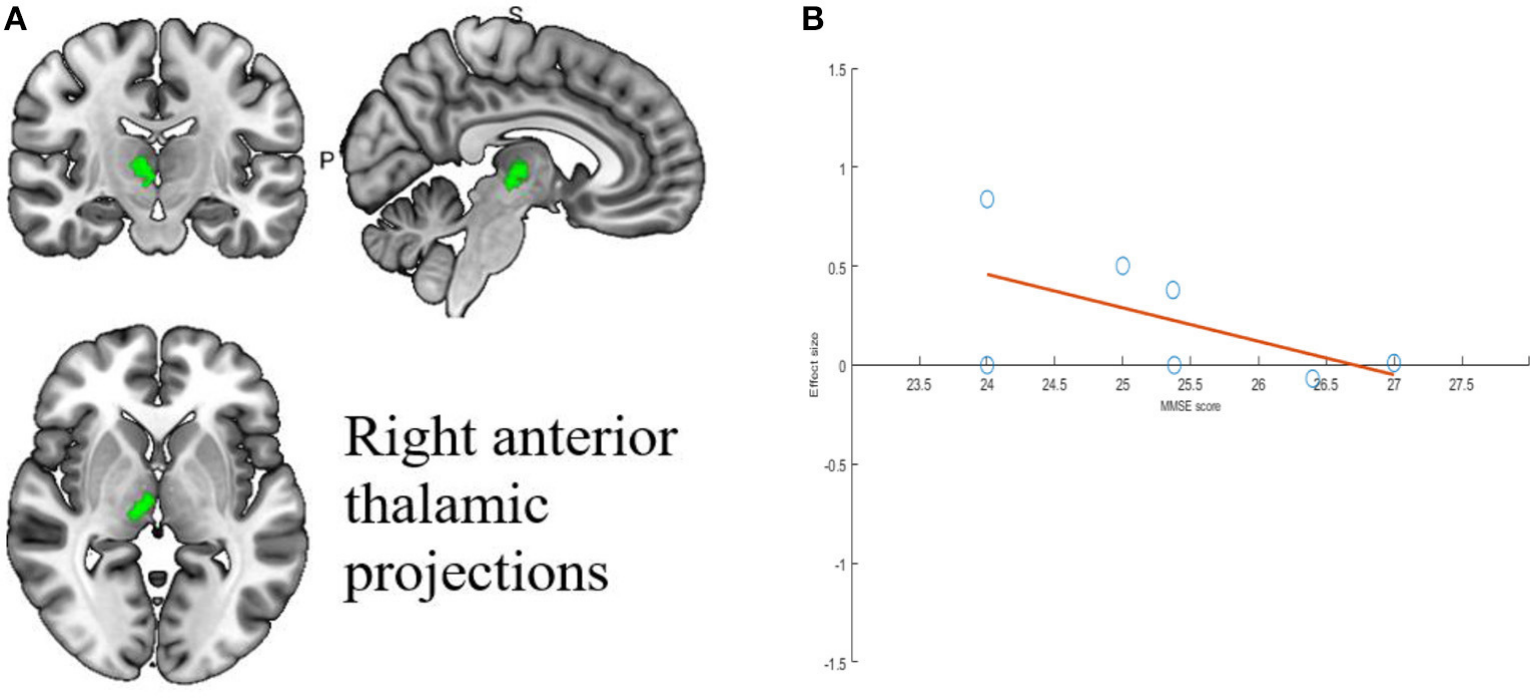
Results of Meta-regression linear model analysis. **(A)** MMSE scores of MCI patients are negatively correlated with regional activity in Right anterior thalamic projections. **(B)** The effect sizes needed to create this plot were extracted from the peak voxels of the maximum slope difference. All studies are indicated by the empty blue circles. Regression lines (Meta-regression SDM slopes) are shown as straight lines.

Meta-regression was used to find potential correlations between acupuncture treatment MCI subjective scale scores, baseline information and brain regions. Whole-brain meta-regression analysis found that MMSE scores in MCI patients were negatively correlated with regional activity in the right corticospinal projection (peak coordinates: *x* = 10, *y* = −18, *z* = −2, voxel = 22, *r* = 0.73, *p* = 0.003354549). However, there were two discrete values in the regression plots. No significant correlation was found between any regional functional change and mean age, gender percentage, education, or CDR score. The meta-regression analysis of brain regions significantly associated with MMSE scores is shown in Table 6 and Figure 5.

**Table 6:** Meta-regression analysis of MMSE scores in treatment group.

**Region**	**MNI** **coordinate**			**SDM** ***z*-score^a^**	***p*-value^b^**	**Number of voxels^c^**
	***x***	***y***	***z***			
Right anterior thalamic projections	8	−18	0	2.622	0.001930118	130

a Peak height threshold: *z* > 1.

b Voxel probability threshold: *p* < 0.005.

c Cluster extent threshold: number ≥ 10 voxels. SDM, signed differential mapping.

## Discussion

In this study, we used the SDM-PSI method to summarize the effect of acupuncture on the modulation of MCI brain regions from the neuroimaging perspective through a meta-analysis of brain imaging studies of MCI patients treated with acupuncture. The within-group comparison results confirmed the effect of acupuncture on regional brain region modulation in MCI patients, with enhanced activity in the right insula, left anterior cingulate/paracingulate gyri, right thalamus, right middle frontal gyrus, right cingulate/central parabrachial gyrus and right middle temporal gyrus brain regions after acupuncture treatment. Further analysis of RCT and longitudinal studies showed that Reho values were significantly elevated in two brain regions, the left anterior cingulate/paracingulate gyrus and the right insula, after acupuncture. Also, the results of intergroup comparison showed significant differences in brain activation regions in the MCI group compared with the healthy control group after acupuncture treatment, mainly in the right supramarginal gyrus. In addition, the right anterior thalamic projection ReHo index was significantly correlated with MMSE scores. Functional characterization showed that these regions were mainly involved in cognitive, emotional, and decision-making regions, which may provide a possible central mechanism for acupuncture treatment of MCI from a neuroimaging perspective.

### Modulatory effects of acupuncture on brain regions of MCI

The results showed that MCI patients had increased Reho values and enhanced brain region activity in the right insula, left cingulate/paracentral gyrus, right thalamus, right middle frontal gyrus, right median cingulate/paracingulate gyri, and right middle temporal gyrus after acupuncture treatment. In particular, activity was significantly increased in the left anterior cingulate/paracingulate gyrus and the right insula. The above brain regions were mainly involved in cognitive, emotional, and decision-making regions, which confirmed to some extent the modulatory effect of acupuncture on brain regions in MCI. Regional homogeneity (ReHo), a measure of local resting functional connectivity, has been shown to be a promising biomarker in a variety of psychiatric disorders (Liu et al., 2008; Chen et al., 2013; Jiang and Zuo, 2016). ReHo is a voxel-based measure of brain activity that assesses the time series of a given voxel in relation to its similarity or synchrony between the time series of a particular voxel (Liu et al., 2008).

A brain imaging study of Parkinson's disease with mild cognitive impairment (PD-MCI) showed reduced ReHo values and reduced spontaneous synchronization in the left insula and that ReHo values were significantly correlated with the Montreal Cognitive Assessment Scale (Li et al., 2020). The association between regional ReHo values and the clinical severity index of cognitive impairment (i.e., MoCA) may laterally validate the potential correlation between the insula and cognitive function. The insula is associated with sensory, motor, visual perceptual, memory, and executive impairments (Chang et al., 2016; Namkung et al., 2018). A functional neuroimaging study of the insula showed that activation of the anterior insula cortex and anterior cingulate cortex was the most common focus of cognitive tasks, including detection processes of perception and consciousness (Sterzer et al., 2007). This study showed that acupuncture therapy was also associated with hyperactivation of the insula and could activate the insula to exert therapeutic effects, which is consistent with previous studies.

The anterior cingulate gyrus (ACC) is a critical limbic system component. Previous studies have shown that ACC is primarily involved in affective motivation and cognitive attention (Bush et al., 2000; Apps et al., 2016). A neuroimaging meta-analysis on MCI showed reduced anterior cingulate ReHo values in amnestic MCI, which could serve as a potential imaging biomarker for MCI and also as a new target for appropriate intervention to delay progression (Song et al., 2021). Previous studies have shown that acupuncture activates brain regions in the anterior cingulate gyrus, primarily in MCI, ischemic stroke, and migraine (Tan et al., 2017; Wu et al., 2018; Chang et al., 2021). Our meta-analysis found that acupuncture increased ReHo in the Left anterior cingulate/paracingulate gyri, which is consistent with the previous findings. Therefore, the increased ReHo of left anterior cingulate/paracingulate gyri may be a potential mechanism for acupuncture in the treatment of MCI. The thalamus, as a diverse hub, is involved in a wide range of behavioral cognitions, such as arousal regulation, attentional selection, and working memory (Saalmann et al., 2012; Crossley et al., 2013; De Bourbon-Teles et al., 2014; Hwang et al., 2017). The thalamus has been shown to interact with different cortical regions convergently and be involved in various cognitive functions, and memory may be the first cognitive function formally associated with the thalamus (Wolff and Vann, 2019). A clinical study on functional magnetic resonance imaging (fMRI) techniques for aMCI showed reduced right thalamic ReHo values in patients with aMCI compared to normal older adults (Min et al., 2019). In a clinical trial of acupuncture in relation to MCI, patients with MCI showed significant changes in functional connectivity in brain regions such as the hippocampus, thalamus and syrinx gyrus after acupuncture to K13 compared to HC (Feng et al., 2012). The thalamus, ACC, and insula constitute significant central autonomic networks, and they are also commonly activated in tasks related to emotion, memory, and mutual sensation (Cauda et al., 2012; Lee et al., 2020). Thus, acupuncture's modulation of brain regions in MCI may be related to homogeneous regional activation of the thalamus, insula and ACC, but the presence of structural-functional connections remains to be further investigated.

The middle frontal gyrus (MFG) is involved in attention, working memory, and language related processing (Briggs et al., 2021). Several functional magnetic resonance imaging studies have shown that the middle frontal gyrus (MFG) plays a role in working memory (Klingberg et al., 1997; Vartanian et al., 2013; Yin et al., 2016). In a study examining the different responses to acupuncture in MCI patients and age-matched healthy individuals as reflected by the regional homogeneity (ReHo) index, the elevated ReHo values in MCI patients were mainly distributed in the middle temporal gyrus (MTG), superior parietal lobe (SPL), middle frontal gyrus (MFG), and superior marginal gyrus (SMG) in the resting state after acupuncture (Liu et al., 2014). Numerous studies have shown that right median cingulate/paracingulate gyri (DCG) is associated with cognitive function, possibly as part of the default network connection (Feng et al., 2019; Cui et al., 2021). A study showed that the integration and dissociation of dynamic functional connectivity states tended to decrease as MCI worsened, and in some states, such as IPL.L-MTG.R and DCG.R-SMG.L, functional brain connectivity was significantly enhanced (Jiao et al., 2021). Reduced glucose metabolism in the right middle temporal gyrus (RMTG) is a powerful biomarker of subjective cognitive decline (Dong et al., 2021). According to previous reports, the MTG region has close functional connectivity with the hippocampus, is primarily involved in verbal or semantic cognition, and is also associated with oral memory (Vandenberghe et al., 1996; Beason-Held et al., 2021). In previous studies, changes in MTG after acupuncture treatment were mainly in Parkinson's Disease (Chae et al., 2009; Yeo et al., 2018). An fMRI study on acupuncture for Parkinson's disease showed increased connectivity between the left MTG and the pre-central gyrus (PCG) in the acupuncture group (Yu et al., 2019). And as a result of dopamine insensitivity, patients with Parkinson's disease have some degree of cognitive deficits (Robbins and Cools, 2014). Also, several studies have shown that there is a co-morbid mechanism between Parkinson's disease and cognitive impairment in neuroimaging, which also provides a reference for future research on the mechanism of acupuncture on cognitive impairment (Delgado-Alvarado et al., 2016; Jozwiak et al., 2017; Baiano et al., 2020).

In addition, the results of the group comparison showed that there was a significant difference between the MCI group in the right supramarginal gyrus after acupuncture treatment compared to the healthy control group. This may imply a specific brain region modulatory effect of acupuncture in the MCI population compared to healthy individuals. In a brain imaging study exploring region-specific neurovascular uncoupling associated with cognitive decline in patients with Parkinson's disease, it was shown that local regulatory abnormalities in the PD-MCI group were specific and restricted to brain regions such as the right supramarginal gyrus and right angular gyrus, which is consistent with the findings of the present study (Shang et al., 2021). Notably, there was a high degree of overlap between the activated brain regions involved in the acupuncture treatment of MCI in the current study and the brain regions where the pain occurred (Henderson et al., 2007; Christidi et al., 2020; Smith et al., 2021). Previous studies have demonstrated that pain processing disorders are often present in patients with cognitive impairment and that various forms and degrees of dementia can affect pain processing (Cole et al., 2006; Kunz et al., 2007, 2009; Jensen-Dahm et al., 2014; Defrin et al., 2015; Beach et al., 2016). A study on pain processing in MCI and its relationship to executive function and memory showed a strong association between pain response and executive function in MCI patients, meaning that poorer executive function was associated with pain onset and escalation (Lautenbacher et al., 2021). This also demonstrates to some extent that the modulatory effect of acupuncture on the brain regions of MCI may also have a modulatory effect on the brain regions of pain, providing a good idea for the multi-target disease treatment of acupuncture.

### Basic characteristics of research on acupuncture for MCI

Of the seven studies included, all were from China, which may be because acupuncture is more popular and widely accepted in Chinese society but can cause language bias. The small sample size of between 22–64 cases per study may be due to the limitations of MRI trials on sample size. Because many functional MRI studies are small and performed at a single site, meta-analyses are thought to help improve the accuracy of results and generalize conclusions from individual studies (Cohn and Decker, 2003). Although the field has studied the optimal sample size needed to detect or evaluate experimental factors, the number of subjects is often limited by practical constraints such as scanning time and cost (Desmond and Glover, 2002; Murphy and Garavan, 2004; Mumford and Nichols, 2008). However, too small a sample size may lead to excessive random errors and make the study results more heterogeneous. Estimates of effect sizes, between-and within-subject variance, and temporal autocorrelation matrices should be added to reduce subject bias's adverse effects on study results (Guo et al., 2014).

Regarding the choice of specific acupuncture modalities, manual acupuncture (MA) and electroacupuncture (EA) are the most common methods used to treat MCI. According to the theory of acupuncture, the stimulation of MA comes from specific finger manipulation that drives the translation, rotation, or tremor of the needle (Dilts et al., 2021). Electroacupuncture works by setting up electrical stimulation at specific points on the body, thereby activating these neural networks and regulating the function of certain organs (Ulloa, 2021). With these two modalities, specific neural network modulation of brain regions can be achieved in MCI patients. LR3 is located between the first and second metatarsal bones on the dorsal side of the foot, in the anterior depression of the metatarsal union, and is part of the Jueyin Liver Meridian of Foot. The K13 point is located on the medial side of the foot, in the depression between the back of the inner ankle and the tendon of the heel bone, and is part of the Shaoyin Kidney Meridian of Foot. Recent studies have shown that acupuncture LR3 and K13 play a positive activating role in social behavior and decision-making in MCI (Chen et al., 2014b). Among the single duration and duration of acupuncture treatment, only one study mentioned that the duration of acupuncture treatment should be 40 min, and most of the studies used a block design to observe the immediate effects of acupuncture. Acupuncture of LR3 and K13 specifically regulates blood flow and activates brain regions associated with emotion, decision making, semantic processing, memory, attention, and sensation. Three studies used only one acupuncture point for treatment, while others combined multiple acupuncture points for MCI. Studies suggest that the combination of acupuncture points may produce novel central effects. Comparison of LR3 plus KI3 acupuncture vs. LR3 alone revealed that ALFF alterations were concentrated in BA6, BA10, BA24, BA32, the posterior cerebellum lobe, and inferior semilunar lobule regions of the brain (Zhang et al., 2016). Studying the combined LR3 and KI3 acupuncture patterns with the relationship between LR3 and KI3 acupoint patterns and regional activation in the brain will be important in the future.

### Study design

The rigorous and scientific clinical trial design is essential to observe the efficacy and effectiveness of interventions. A rigorous and scientific clinical trial design is essential to observe the efficacy and effectiveness of an intervention. In this study, three clinical trial designs including randomized controlled trials (RCTs), cross-sectional studies, and longitudinal studies were included, and the results of the meta-analysis of brain images between different study designs were also presented. RCTs are considered the highest level of evidence to establish causal associations in clinical research (Zabor et al., 2020). Limitations in the case-control study design used in most clinical fMRI studies tend to raise questions as to how samples are drawn and matched to potentially confounding variables (Carter et al., 2008). In contrast, only one of the original studies we included was an RCT, and most were cross-sectional studies. Currently, the application of MRI modalities has shown promising results in cross-sectional studies of neurodegenerative diseases (Argiris et al., 2021). However, in studies related to cognitive function, cross-sectional showed differences in outcomes between cross-sectional and longitudinal, as aptly demonstrated by the results of the meta-analysis in this study (Salthouse, 2019).

In most of our studies, the control group was healthy individuals, and only three studies used the placebo group as a control group. Randomized controlled trials with placebo control groups have high internal validity and are considered a reliable method for assessing treatment effects (Gøtzsche, 1994; Walach and Loef, 2015). The long time required for MRI acquisition resulted in the inability to set up a placebo control group due to the non-participation of many potential subjects, ultimately creating a potential bias (Carter et al., 2008). In this study, a placebo control group was set up for sham acupuncture. Sham acupuncture (SA), also known as a placebo, may be considered a sham intervention because it is based on non-acupuncture points. “Sham acupuncture” controls, in which needles are inserted at wrong points or non-points, which deliberately violate traditional acupuncture theories of point locations or indications and are therefore predicted to be incapable of achieving the outcomes intended by true acupuncture (Moffet, 2009). In clinical trials, the placebo control group should be consistent with the treatment group at baseline, except for physiological inertia (Chae, 2017). Currently, relevant validation focuses only on the blinding and credibility of the interventions, and few studies have validated the physiological inertia of these sham interventions. Sham acupuncture has a non-inert character, which can cause the public to question its actual effectiveness. In addition, in acupuncture trials, the key to distinguishing between acupuncture and sham acupuncture is the presence or absence of the sensory stimulus of “getting qi”. Data from imaging studies also suggest that expectation, learning, and contextual factors play an important role in the placebo effect (Enck et al., 2008; Wager and Atlas, 2015; Geuter et al., 2017).

### Experimental designs of MRI

The rs-fMRI and the task-state fMRI are the two primary paradigms for functional MRI studies. In the experimental design of MRI on acupuncture, rs-fMRI is the closest to the response of brain activity in the actual state, while task-state fMRI reflects the persistent effect of acupuncture. In the present study, a non-repeated event-related block design was mainly used, in which the inserted needles were continuously stimulated for 30 s to 2 min before the scan to observe the immediate effects of acupuncture. According to TCM theory, acupuncture produces a sustained effect, even after 30 min of retention, with corresponding neural responses, so NRER is more consistent with the MRI experimental design of acupuncture (Cho et al., 1998; Bai et al., 2009, 2010). They also can reduce interference from the persistent effect of acupuncture that occurs when a single, prolonged acupuncture stimulation is given during the scanning process (Liu et al., 2009). However, this type of experiment usually selects single acupuncture point, which has the limitation of single stimulation to some extent, and the clinical treatment for MCI usually uses multiple acupuncture points.

In recent years, rs-fMRI has provided new research perspectives on the central mechanisms of acupuncture treatment. By observing the changes in ReHo/ALFF after acupuncture, the changes in brain function after acupuncture treatment are analyzed, and such changes are more reflected as long-term cumulative effects. In addition, in recent years, rs-fMRI imaging has been increasingly used to explore the central mechanisms of acupuncture treatment, such as pain, migraine, and stroke (Lan et al., 2013; Chen et al., 2014a; Leung et al., 2014). Therefore, future studies should focus on the experimental design of rs-fMRI as a way to observe the long-lasting therapeutic effects of acupuncture on MCI in the real world.

### Limitations

Although this review provides an SDM-PSI-based meta-analysis of current MRI studies of acupuncture for MCI, there are still some limitations. First, all of the literature that met the inclusion criteria was conducted in China in this study. The language of publication included only Chinese and English, which may lead to potential publication bias and reduce the applicability and readability of this study. Although our search strategy appears to be comprehensive, the possibility of relevant literature appearing in other databases cannot be excluded. The high level of the review emphasizes the need for multi-center studies that especially considering the apparent heterogeneity of social backgrounds between countries (Ewers et al., 2015). Future studies should consider the need for multicenter studies in which language, country, region, and other influencing factors are taken into account to expand treatment coverage. Second, due to the small sample size of the included studies, there is significant heterogeneity between studies in terms of acupuncture point selection, clinical protocol design, and analytical methods, which significantly reduces the credibility of the findings. Given the financial burden and additional limitations of neuroimaging studies, small samples are standard, which can lead to low statistical power and may obscure essential results that may be clinically significant (Moayedi et al., 2018). Therefore, more rigorous randomized controlled trials with large samples should be designed to avoid confounding factors and methodological bias in future clinical trials.

In order to make the findings more reproducible and accurate and to precisely elucidate the mechanisms of brain region regulation in acupuncture for MCI, larger sample sizes of RCTs are needed in the future. When designing and reporting MRI for acupuncture studies, investigators should follow the Standards for Reporting Interventions in Controlled Trials of Acupuncture (STRICTA) guidelines (MacPherson et al., 2010). Standardized clinical treatment protocols should be developed around six regions: the theoretical rationale for acupuncture, details of acupuncture measures, treatment protocols, ancillary interventions, acupuncturist credentials, and control interventions. Third, most of the studies did not specify the specific implementation of blinding and allocation concealment in the clinical trial design, which exposes the results to a certain degree of risk of methodological bias. In addition, most of the studies had healthy controls, making it difficult to exclude the placebo effect of acupuncture or other factors from interfering. Future studies should design rigorous scientific RCTs with sham acupuncture placebo controls to increase the reliability of MRI mechanism studies. In subsequent clinical designs, the design of sham acupuncture groups should be standardized, and more standard implementation guidelines should be adopted for appliance selection, baseline patient characteristics, and effect determination to maximize the actual therapeutic effect of acupuncture (Birch et al., 2022). Fourth, adverse events during acupuncture treatment and MRI acquisition were not reported in any studies. We suggest that in future studies and reporting on the primary outcome, clinical changes and adverse events during treatment should be monitored simultaneously to provide comprehensive standardized clinical guidelines for neuroimaging studies.

### Future outlook of acupuncture for MCI

Early screening for MCI is generally performed through cognitive assessments, such as the Montreal Cognitive Assessment (MoCA), MMSE, etc (Gauthier et al., 2006). However, the accuracy of MCI diagnosis is compromised by the highly subjective nature of cognitive assessment and its low sensitivity to early identification of MCI and dementia. Some studies in which neuroimaging may help determine the etiology and prognosis of MCI suggest that structural magnetic resonance imaging (MRI), Fludeoxyglucose PET, and other neuroimaging may help identify people with MCI and those at high risk of progressing from MCI to dementia (Langa and Levine, 2014). Most recently, PET imaging of the extent of Aβ plaques in the brain has become more feasible with the radiopharmaceutical tracer florbetapir (Clark et al., 2011). Currently, amyloid A (Chen et al., 2021). Likewise, the efficacy and effectiveness of acupuncture can be evaluated similarly. In addition to the use of MRI to investigate the central mechanisms of acupuncture, quantitative analysis of efficacy can be performed using biomarkers based on PET technology. In addition, indicators of cognitive function, functional status, medications, neurological or psychiatric abnormalities, and laboratory tests are combined to distinguish MCI from normal aging or dementia and identify possible forms of mild cognitive impairment caused by other conditions. Older adults fear cognitive decline, and most patients prefer testing that would indicate future Alzheimer's disease risk (Wikler et al., 2013). Clinical prediction and management of MCI may become a hot topic in the future as diagnostic techniques change. Recently, Yang et al. developed a clinical prediction model for acupuncture treatment in patients recovering from stroke under different conditions by standardizing acupuncture treatment in 1,410 patients recovering from a stroke, combined with CART decision tree analysis, to provide a tool for predicting the effect of acupuncture treatment (Burge et al., 2014). Regarding the prediction model of MCI, the current research directions are primarily focused on the conversion of mild cognitive impairment to Alzheimer's disease. For example, Huang et al., based on the Least Absolute contraction and Selection Operator (LASSO), which provides a personalized MCI to AD, found significant associations between neuropsychological scores, cortical features, Aβ levels, and underlying genetic pathways (Huang et al., 2020). The above study shows that the establishment of a neuroimaging-based prediction model can guide clinical diagnosis and provide an objective reference for the assessment of the efficacy of acupuncture in the treatment of MCI and contribute to the development of personalized treatment. In addition, the prediction of acupuncture response can reduce the medical costs for patients identified as likely non-responders. Future research can start from the prediction model of acupuncture for MCI and integrate machine learning algorithms better to explain the neuroimaging mechanism of acupuncture for MCI.

## Conclusion

A ReHo-based meta-analysis showed that acupuncture has potential modulatory effects on MCI in brain regions, suggesting that the left anterior cingulate/paracingulate gyrus, right insula, right thalamus, right middle frontal gyrus, right median cingulate/paracingulate gyri, and right middle temporal gyrus may be the precise brain region response targets of acupuncture for MCI. In particular, activity was significantly increased in the left anterior cingulate/paracingulate gyrus and the right insula. The MCI group showed stronger activity in the right supramarginal gyrus after acupuncture treatment compared to healthy controls. The above study provides a new perspective to elucidate the role of brain region modulation in acupuncture for MCI. In the future, guidelines and procedures should be followed to conduct large-scale rigorous randomized controlled trials to ensure the validity of future clinical evidence.

## Data availability statement

The original contributions presented in the study are included in the article/Supplementary material, further inquiries can be directed to the corresponding author/s.

## Author contributions

SM and HH designed the entire study and wrote the manuscript. ZZ and HZ screened the study for inclusion in the study. ML and LY performed the data extraction. SM and BY were involved in the analysis of the data. HW made good suggestions for the article. All authors read and approved the final manuscript.

## Funding

This work was supported by National Natural Science Foundation of China (Grant No. 82074548).

## Conflict of interest

The authors declare that the research was conducted in the absence of any commercial or financial relationships that could be construed as a potential conflict of interest.

## Publisher's note

All claims expressed in this article are solely those of the authors and do not necessarily represent those of their affiliated organizations, or those of the publisher, the editors and the reviewers. Any product that may be evaluated in this article, or claim that may be made by its manufacturer, is not guaranteed or endorsed by the publisher.
