# Supplementary material for: Effect of acupuncture on brain regions modulation of mild cognitive impairment: A meta-analysis of functional magnetic resonance imaging studies
Source: Front Aging Neurosci. 2022 Sep 23;14:914049. doi: 10.3389/fnagi.2022.914049 (PMC9540390; doi:10.3389/fnagi.2022.914049)
Supplement: Supplementary file 1 [file Data_Sheet_1.docx]

**Supplementary Materials**

**TableS1. Checklist of the PRISMA**

| **Section/topic** | **#** | **Checklist item** | **Reported on page #** |
| --- | --- | --- | --- |
| **TITLE** | | |  |
| Title | 1 | Identify the report as a systematic review, meta-analysis, or both. | 1 |
| **ABSTRACT** | | |  |
| Structured summary | 2 | Provide a structured summary including, as applicable: background; objectives; data sources; study eligibility criteria, participants, and interventions; study appraisal and synthesis methods; results; limitations; conclusions and implications of key findings; systematic review registration number. | 1-2 |
| **INTRODUCTION** | | |  |
| Rationale | 3 | Describe the rationale for the review in the context of what is already known. | 1-2 |
| Objectives | 4 | Provide an explicit statement of questions being addressed with reference to participants, interventions, comparisons, outcomes, and study design (PICOS). | 3-4 |
| **METHODS** | | |  |
| Protocol and registration | 5 | Indicate if a review protocol exists, if and where it can be accessed (e.g., Web address), and, if available, provide registration information including registration number. | 4 |
| Eligibility criteria | 6 | Specify study characteristics (e.g., PICOS, length of follow-up) and report characteristics (e.g., years considered, language, publication status) used as criteria for eligibility, giving rationale. | 5 |
| Information sources | 7 | Describe all information sources (e.g., databases with dates of coverage, contact with study authors to identify additional studies) in the search and date last searched. | 4 |
| Search | 8 | Present full electronic search strategy for at least one database, including any limits used, such that it could be repeated. | 4 |
| Study selection | 9 | State the process for selecting studies (i.e., screening, eligibility, included in systematic review, and, if applicable, included in the meta-analysis). | 5 |
| Data collection process | 10 | Describe method of data extraction from reports (e.g., piloted forms, independently, in duplicate) and any processes for obtaining and confirming data from investigators. | 5-6 |
| Data items | 11 | List and define all variables for which data were sought (e.g., PICOS, funding sources) and any assumptions and simplifications made. | 6 |
| Risk of bias in individual studies | 12 | Describe methods used for assessing risk of bias of individual studies (including specification of whether this was done at the study or outcome level), and how this information is to be used in any data synthesis. | 6 |
| Summary measures | 13 | State the principal summary measures (e.g., risk ratio, difference in means). | 6-7 |
| Synthesis of results | 14 | Describe the methods of handling data and combining results of studies, if done, including measures of consistency (e.g., I^2^) for each meta-analysis. | 6-7 |

Page 1 of 2

| **Section/topic** | **#** | **Checklist item** | **Reported on page #** |
| --- | --- | --- | --- |
| Risk of bias across studies | 15 | Specify any assessment of risk of bias that may affect the cumulative evidence (e.g., publication bias, selective reporting within studies). | 7 |
| Additional analyses | 16 | Describe methods of additional analyses (e.g., sensitivity or subgroup analyses, meta-regression), if done, indicating which were pre-specified. | 7 |
| **RESULTS** | | |  |
| Study selection | 17 | Give numbers of studies screened, assessed for eligibility, and included in the review, with reasons for exclusions at each stage, ideally with a flow diagram. | 7 |
| Study characteristics | 18 | For each study, present characteristics for which data were extracted (e.g., study size, PICOS, follow-up period) and provide the citations. | 7-8 |
| Risk of bias within studies | 19 | Present data on risk of bias of each study and, if available, any outcome level assessment (see item 12). | 8 |
| Results of individual studies | 20 | For all outcomes considered (benefits or harms), present, for each study: (a) simple summary data for each intervention group (b) effect estimates and confidence intervals, ideally with a forest plot. | 8 |
| Synthesis of results | 21 | Present results of each meta-analysis done, including confidence intervals and measures of consistency. | 8 |
| Risk of bias across studies | 22 | Present results of any assessment of risk of bias across studies (see Item 15). | 9 |
| Additional analysis | 23 | Give results of additional analyses, if done (e.g., sensitivity or subgroup analyses, meta-regression [see Item 16]). | 9 |
| **DISCUSSION** | | |  |
| Summary of evidence | 24 | Summarize the main findings including the strength of evidence for each main outcome; consider their relevance to key groups (e.g., healthcare providers, users, and policy makers). | 9-10 |
| Limitations | 25 | Discuss limitations at study and outcome level (e.g., risk of bias), and at review-level (e.g., incomplete retrieval of identified research, reporting bias). | 14 |
| Conclusions | 26 | Provide a general interpretation of the results in the context of other evidence, and implications for future research. | 16 |
| **FUNDING** | | |  |
| Funding | 27 | Describe sources of funding for the systematic review and other support (e.g., supply of data); role of funders for the systematic review. | 17 |

*From:*  Moher D, Liberati A, Tetzlaff J, Altman DG, The PRISMA Group (2009). Preferred Reporting Items for Systematic Reviews and Meta-Analyses: The PRISMA Statement. PLoS Med 6(6): e1000097. doi:10.1371/journal.pmed1000097

For more information, visit: **www.prisma-statement.org**.

**S2. Anatomical localization of the acupoints mentioned in the included studies**

1.DU20(Baihui):It is located at the intersection of the median line at the top of the head and the line connecting the tips of the ears.

2.BG13(Benshen):0.5 cun above the front hairline, 3 cun next to the Shenting point.

3.GB20(Fengchi):The back of the human neck, 1 cun straight up from the midline of the posterior hairline, directly under the external occipital ridge, between the sternocleidomastoid muscle and the upper end of the trapezius muscle.

4.LR3(Taichong):It is located on the dorsal side of the foot, in the depression before the union of the first and second metatarsals.

5.SP3(Taibai):The depression on the medial edge of the sole of the foot, just below the posterior aspect of the first metatarsal tuberosity.

6.KI3(Taixi):In the ankle area, the depression between the tip of the inner ankle and the Achilles tendon.

7.RN4(Guanyuan):In the lower abdomen, on the anterior median line, 3 inches below the middle of the umbilicus.

8.HT7(Shenmen):It is located in the wrist, at the ulnar end of the transverse wrist palmar line, at the radial recess of the ulnar carpal flexor tendon.

9.ST40(Fenglong):On the anterolateral side of the lower leg, when the tip of the outer ankle is 8 cun above, two fingers from the front edge of the tibia.

10.BL58(Feiyang):It is located on the posterior and lateral side of the lower leg, 7 cun straight up from the midpoint of the horizontal line between the tip of the outer ankle and the Achilles tendon, when the posterior edge of the fibula is located.

11.LI4(Hegu):On the dorsum of the hand, between the 1st and 2nd metacarpals, when the midpoint of the radial side of the 2nd metacarpal.

12.EX-HN1(Sishencong):Located at the top of the head in front, behind, to the left and right of the Baihui point 1 cun away from each side, a total of 4 points.

13.ST36(Zusanli):On the lateral side of the lower leg, 3 cun below the Dubi point, on the line between the Dubi point and the Jiexi point.

14.SP6(Sanyinjiao):On the medial side of the lower leg, when the tip of the inner ankle of the foot is 3 cun above, behind the medial edge of the tibia.

Note: "cun" is the traditional Chinese unit of length, 1 cun = 3.33 cm
